# Construction and application of the core competence course training system for infectious disease specialist nurses

**DOI:** 10.1186/s12909-024-05405-2

**Published:** 2024-04-15

**Authors:** Chao Wu, Hongli Zhang, Yawei Lin, Weiyun Yuan, Jing He, Lu Li, Donglei Jiang, Zhaohua Ji, Hongjuan Lang

**Affiliations:** 1 Nursing Department, Air Force Medical University, No. 169 Changle West Road, 710032 Shaanxi, Shaanxi China; 2 School of Nursing, Shaanxi University of Chinese Medicine, Xianyang, China; 3 Department of Anesthesiology, 956Th Hospital of the Chinese People’s Liberation Army, Nyingchi, China; 4 Xijing Hospital of Air Force Medical University, Shaanxi, China; 5 Laboratory Department, Yan’an University Affiliated Hospital, Yan’an, China; 6 Tangdu Hospital of Air Force Medical University, Shaanxi, China; 7 Department of Foreign Languages, School of Basic Medicine, Air Force Medical University, No. 169 Changle West Road, 710032 Shaanxi, Shaanxi China; 8 Department of Epidemiology, Ministry of Education Key Lab of Hazard Assessment and Control in Special Operational Environment, School of Public Health, Air Force Medical University, Shaanxi, China

**Keywords:** Training course system, Infectious disease specialist nurses, Core competence, Delphi study, Empirical research

## Abstract

**Objectives:**

This study aims to construct and apply a training course system which was scientific and comprehensive to foster the core competence of infectious disease specialist nurses.

**Design:**

A two-round Delphi consultation survey was carried out to collect feedback from experts on constructing the training course system of core competence for infectious disease specialist nurses. Besides, a non-randomized controlled experimental study was adopted to check the application effect of the courses.

**Methods:**

This study adopted a series of methods including group discussion, theoretical analysis and Delphi consultation to draft the training course content of core competence of infectious disease specialist nurses. Twenty-one Chinese experts were invited to participate in the Delphi consultation from November 2021 to December 2021. From October 2022 to January 2023, a total of 105 infectious disease specialist nurses from two training bases were selected by the convenience sampling method, of which the nurses in one training base were the control group and the nurses in the other training base were the observation group. The observation group was trained by the constructed core competence training course. Questionnaire evaluation was used to compare the core competence of infectious disease specialist nurses and the training effect.

**Results:**

The experts, regarded as the authorities on the subject, were highly motivated in this study. Besides, they reached a consensus on the results. The final training course system of core competence for infectious disease specialist nurses focused on 5 competence modules and was composed of 12 categories of courses with 66 classes and corresponding objectives. The core competence scores of the observation group were significantly higher than those in the control group after training (*P* < 0.05), which proved the training system can effectively enhance the core competence of infectious disease specialist nurses.

**Conclusions:**

The research methods embodied scientific and precise properties. The course system was comprehensive in content and reliable in results. It could serve as a reference for training infectious disease specialist nurses.

## Introduction

With the rapid development of medical treatment, especially in the era of knowledge, nurses must continue to learn in order to meet the needs of professional development [1, 2]. It is reported that continuing education and vocational learning are the main ways for nurses to acquire knowledge [3]. Continuing education and scientific training can help post-qualification nurses to master new knowledge [4, 5]. Specialist nurses refer to expert clinical nurses with high competence and expertise in a specialized nursing field, who can use their knowledge, expertise and technology to provide nursing services for patients and social groups [6, 7]. As the backbone of the nursing team, specialist nurses play a critical role in team dynamics [8, 9]. Therefore, continuing education for this group is particularly important. In China, however, infectious disease specialist nurse training and infection control education are still in the initial stage, we need to further optimize the teaching arrangements, teaching methods and training procedures [10–12]. Since nurse specialization is a field that does not share the same standardization as medical specialties do, a lot of heterogeneity exists.

Infection prevention has always been a very important field even before the COVID-19 pandemic. It is essential for preventing healthcare-associated infections, and particularly the spread of multidrug resistant bacteria [13–16]. Nurses’ infection prevention and infection nursing ability not only guarantees their own safety, but also greatly affects the quality and efficiency of infectious disease nursing. As an important way of cultivation, nursing training ensures the sustainability of the nursing team [17]. Therefore, how to train infectious disease specialist nurses effectively to meet the practical needs is of great importance and urgency. More and more studies focus on the ability of infectious disease nurses [18–20]. But it is noticeable that there are no mature, systematic and scientific training courses for Chinese infectious disease specialist nurses. In fact, the training of Chinese specialist infectious disease professionals has been carried out in the training base established by different hospitals, which has then been assessed by their own standards. After passing the assessment, trainees will be awarded the qualification. Owing to the different training standards at individual hospitals, the curriculum, training materials and methods are not uniform. Besides, the medical development in different regions of China is unbalanced. It is not conducive to the homogeneous development of infectious disease specialist nurses. Taking the frequent occurrence of emerging infectious diseases into consideration, the existing training system of infectious disease nursing cannot meet the urgent clinical needs for the development of nursing. The implementation of a standardized curriculum may be the prerequisite for comprehensively improving the core competence of infectious disease nurses, with the courses being the training core.

Therefore, our study aims at constructing and applying a set of scientific training courses for infectious disease specialist nurses which can regulate the training of infectious disease specialist nurses in different hospitals, improve the training quality and enhance the development of infectious disease specialist nursing so as to better respond to the infectious diseases.

## Method

### Construction of a core competence course training system for infectious disease specialist nurses

#### Establishment of the research group

The research group was set up before the research, including one professor, one associate professor, two lecturers, one nurse-in-charge and one postgraduate. The draft of the course training system was constructed on the basis of the index system of core competence assessment for infectious disease specialist nurses constructed in the early stage [19]. The research group analyzed and categorized all the indicators and translated them into competence modules, course categories and class contents, and then compiled the expert consultation questionnaires, distributed the questionnaires and analyzed the feedback.

#### Selection of the experts

We developed a pool of potential participants who met our inclusion criteria and had expertise in infection control. By using the random sampling method and sending invitation, 21 experts out of the expert resources were selected to engage in the consultation. The research group explained the research contents and purposes to the experts before the consultation. Besides, consultation would not be conducted until the consent was got from each expert. The expert panel contained three types of professionals and the inclusion criteria were as follows: (a) for experts on infectious disease health care: they should be engaged in infectious disease health care or infectious disease medical supervision for more than 15 years, obtain the intermediate or advanced level of certificate, have a bachelor degree or above, sign the informed consent form and voluntarily participate in this study; (b) for experts on infectious disease nursing: they should be engaged in infectious disease nursing or infectious disease nursing supervision for more than 15 years, obtain the intermediate or advanced level of certificate, have a bachelor degree or above, sign the informed consent form and voluntarily participate in this study; (c) for experts on infectious disease nursing education, they should be engaged in the teaching of infectious disease nursing in the training class of infectious disease specialist nurses for more than 3 years or in institutions of higher education for more than 15 years, obtain the intermediate or advanced level of certificate, have a bachelor degree or above, sign the informed consent form and voluntarily participate in this study.

#### Construction of the expert consultation questionnaire

The expert consultation questionnaire was composed of three parts: (a) basic information of the experts which included name, age, professional title, years of engaging in infectious disease specialist nurse training and working unit, etc.; (b) the first draft of the course training system of core competence of infectious disease specialist nurses consisting of 5 competence modules, 12 course categories, 46 class contents and their corresponding objectives. The importance of course system was evaluated by way of Likert 5-level scoring method, 5 = very important, 4 = important, 3 = general, 2 = unimportant, 1 = completely unimportant, and the column for suggestions and supplements was provided; (c) expert familiarity with content of the survey and judgement.

#### Questionnaire distribution and analysis

The consultation questionnaires were sent via e-mail. To ensure the quality of the consultation questionnaire, the experts had two weeks to fill in the questionnaire. We revised the questionnaire according to the results of the first-round consultation and issued the revised questionnaire to the experts for the second round. Two rounds of Delphi consultation were carried out during November and December of 2021. In the process, the training courses were refined and revised until the final version of the training course system of core competence for infectious disease specialist nurses was eventually formed.

The statistical software SPSS26.0 was applied in the process of data analysis. Variable distribution was checked for continuous variables and was parametrically distributed variables. Data measurement and data calculation were expressed in the form of mean ± standard deviation, frequency, and percentage respectively. The enthusiasm of the experts was also expressed in the form of questionnaire response rate. The authority degree of the expert opinion was shown by the authority coefficient. The coordination degree of expert opinion was displaced by variable coefficient and Kendall harmony coefficient. It indicated that the difference was statistically significant (*P* < 0.05).

#### Quality control

The research group selected experts from 5 different provinces and cities to participate in the consultation. And the experts were from 3 different fields, namely infectious disease health care, infectious disease nursing and infectious disease nursing education.

### Application of the core competence course training system for infectious disease specialist nurses

#### Participants

A non-randomized controlled experimental study design was adopted in our research and the required sample size was calculated by software G*Power 2.1. Repeated measures analysis of variance statistic method was chosen to estimate the sample size, and with the effect size being 0.8, α being 0.05, power being 0.95, allocation ratio being 1, the total sample size was at least 84.

From October 2022 to January 2023, a total of 105 infectious disease specialist nurses from two training bases were selected by convenience sampling method, the selected nurses in one training base were the control group (50) while the selected ones in the other training bases (55) were the observation group.

#### Research implementation

In the empirical study, the control group did not apply the core competence course training system, and they only participated in the relevant traditional training courses, while the observation group was trained with the core competence course training system.

#### Evaluation methodology

Core competence of infectious disease specialist nurses is measured by self-report questionnaire measurement. We used the infectious disease specialist nurses’ core competence scale [21] which was specifically developed by the research group in the early stage to evaluate their core competence before and after the training. The scale has good reliability and validity. It contained 5 dimensions and 34 items: Professional Development Abilities (11 items), Infection Prevention and Control Abilities (9 items), Nursing Abilities for Infectious Diseases (6 items), Professionalism and Humanistic Accomplishment (5 items) and Responsiveness to Emergency Infectious Diseases (3 items). The scale adopted the 5-level scoring method, ranging from “fully consistent with the item” to “completely disagree with the item”, the scale was scored from “5 points” to “1 point”. The higher the score of the questionnaire was, the higher the infectious disease specialist nurses’ core competence was.

**Fig. 1:**
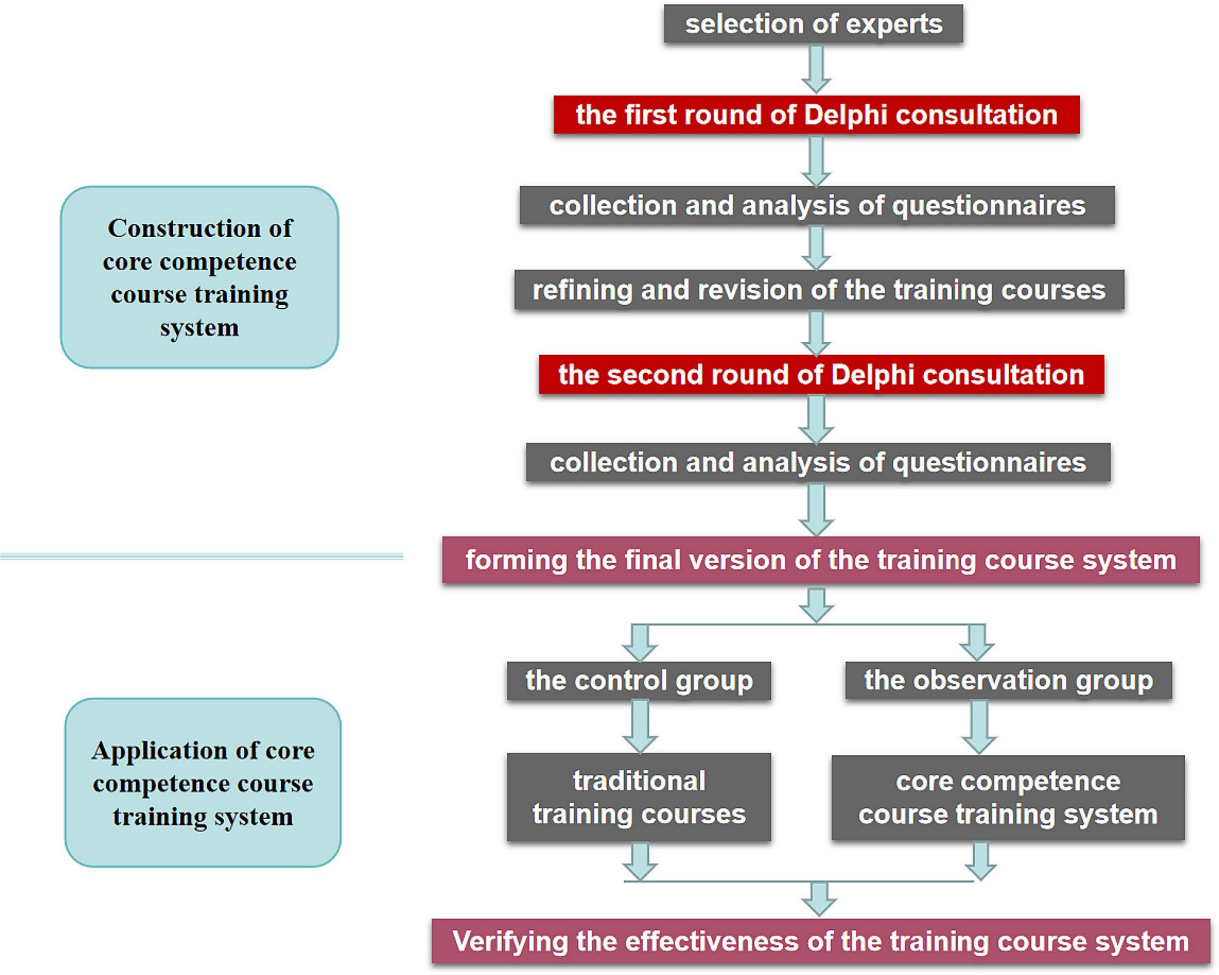
The entire process of the study

From October 2022 to January 2023, before the start of the study and after the implementation of the course, we distributed questionnaires to the two groups of infectious disease specialist nurses to investigate their core competence. After we collected the questionnaire, the differences in the core competence of the two groups before and after training were identified. The entire process was shown in Fig. 1.

#### Qualifications of lecturers

In order to ensure the quality of training, lecturers needed to have the following qualifications: obtaining a bachelor degree or above, having intermediate or above professional titles. When the course content was settled, qualified lecturers were selected and given half a year to prepare for the lectures before they carried out the training.

#### Ethical consideration

This study complied with the Declaration of Helsinki [22, 23]. The purpose and significance of the study were explained to the participants and informed consent of participants was obtained. During the investigation, the participants could terminate and withdraw from the consultation at any time. And their personal information was completely confidential. The inquiry data was only used for this study and not open for other purposes.

## Results

### Construction of a core competence course training system for infectious disease specialist nurses

#### Basic information of the experts

The age of the experts ranged from 34 to 51 years old, with an average of 42.48 (SD 4.63). Their length of service varied from 3 to 24 years, with an average of 14.05 (SD 6.36) years. The details were shown in Table 1.

**Table 1:** Demographic information of experts (*n* = 21)

Item	Project	Frequency	Proportion(%)
Age (years)	<40	5	23.81
	40–49	14	66.67
	≥ 50	2	9.52
Work experience (years)	<15	5	23.81
	15–19	13	61.90
	≥ 20	3	14.29
Research field	Infectious disease health care	4	19.05
	Infectious disease nursing	7	33.33
	Nursing education	10	47.62
Job title	Senior-level	3	14.29
	Associate-senior level	9	42.86
	Intermediate level	9	42.86
Educational background	Doctorate	4	19.05
	Master	12	57.14
	Bachelor	5	23.81
Region	Shaanxi province	7	33.33
	Hubei province	4	19.05
	Zhejiang province	4	19.05
	Beijing city	3	14.29
	Chongqing city	3	14.29

#### Experts’ interest in the topic

The enthusiasm of the experts was assessed on the basis of the return rate of the questionnaires. In this study, during the two rounds of consultation, 21 questionnaires were distributed and 21 valid questionnaires were returned with a response rate of 100%. The details were shown in Table 2.

**Table 2:** Response rate of the questionnaire

Field of the experts	First round			Second round		
	Number of questionnaires distributed	Number of response(ratio)	Proposed suggestion number (ratio)	Number of questionnaires distributed	Number of response(ratio)	Proposed suggestion number (ratio)
Health care	4	4(100%)	2(9.52%)	4	4(100%)	1(4.76%)
Nursing	7	7(100%)	6(28.57%)	7	7(100%)	1(4.76%)
Nursing education	10	10(100%)	10(100%)	10	10(100%)	2(9.52%)
Total	21	21(100%)	18(85.71%)	21	21(100%)	4(19.05%)

#### Experts’ authority coefficient

The authority of the expert opinions was illustrated by the authority coefficient. It was the arithmetic average of the judgement coefficient and familiarity coefficient. The authority coefficient over 0.7 indicated that the result of consultation was reliable. The experts’ judgement was based on practical experience, theoretical analysis, domestic and foreign reference materials and intuitive perception. The degree of expert familiarity was divided into four categories, and each item was referred to a different value shown in Tables 3 and 4.

**Table 3:** Judgment criteria

Judgment criteria	High	Medium	Low
Practical experience	High(0.5)	Medium (0.4)	Low(0.3)
Theoretical analysis	High(0.3)	Medium (0.2)	Low(0.1)
Reference	High(0.1)	Medium (0.1)	Low(0.1)
Intuitive perception	High(0.1)	Medium (0.1)	Low(0.1)

**Table 4:** Experts’ familiarity

Degree of familiarity	Very familiar	Relatively familiar	Familiar	Quite familiar	Completely not familiar
Value	0.9	0.7	0.5	0.3	0.1

In this study, the experts exhibited a judgement coefficient of 0.952, a degree of expert familiarity of 0.962, and an authority coefficient of 0.957, as evidenced in Tables 5 and 6.

**Table 5:** Frequency of experts’ judgement

Judgment criteria	High		Medium		Low	
	Numbers	Frequency(%)	Numbers	Frequency(%)	Numbers	Frequency(%)
Practical experience	14	66.67	7	33.33	0	0
Theoretical analysis	18	85.71	3	14.29	0	0
Reference	5	23.81	10	47.62	6	28.57
Intuitive perception	14	66.67	5	23.81	2	9.52

**Table 6:** Frequency of experts’ familiarity

Degree of familiarity	Very familiar		Relatively familiar		Familiar		Quite familiar		Completely not familiar	
	Numbers	frequency	Numbers	frequency	Numbers	frequency	Numbers	frequency	Numbers	frequency
Self-assessment	17	80.95	4	19.05	0	0	0	0	0	0

#### Experts’ opinion coordination degree

The experts’ opinion coordination degree was used to judge whether the experts had disagreements on the importance of the items. It was normally expressed by Kendall’s coefficient of concordance (Kendall’s W). The Kendall’s W ranged from 0 to 1. The closer to 1 it was, the higher the degree of experts’ opinion coordination was.

In the first round of consultation, the Kendall’s W of competence module, course category and class content were 0.280, 0.292 and 0.303 respectively while in the second round of consultation, the data were 0.301, 0.350 and 0.253 respectively. The Kendall’s W test showed statistical significance (*P*<0.05), indicating that the experts’ opinion coordination degree in the two rounds of consultation was satisfactory, as shown in Table 7.

**Table 7:** Experts’ coordination degree

Round	Index	Number	Kendall’s W	χ^2^	P
The first round	Competence module	5	0.280	23.520	0.000
	Course category	12	0.292	67.357	0.000
	Class content	46	0.303	286.604	0.000
The second round	Competence module	5	0.301	25.250	0.000
	Course category	12	0.350	80.837	0.000
	Class content	66	0.253	341.092	0.000

#### Results of the Delphi consultation

In the first round of questionnaire consultation, 18 experts put forward their suggestions on the revision of the course system. After group discussion, we modified the course system: revising 2 course categories, deleting 1 class content, revising 10 class contents, adding 10 class contents, splitting 1 class content into 2 parts, revising 12 class objectives and adding 22 class objectives. The specific modifications were as follows. II-1 Introduction to Infectious Diseases and II-2 Responding to Infectious Diseases were respectively revised to II-1 The ability of predicting epidemics and conducting emergency drills and II-2 The ability of responding to infectious disease epidemics. I-1-3 Questionnaire designing and data collection was revised to How to prepare questionnaire and select data. I-1-5 Common medical statistical approaches was revised to Medical statistics. I-3-1 Good curriculum design was revised to How to design classes and conduct teaching methodology. II-1-3 Emergency plan and response procedures for public health emergencies of the infectious diseases was revised to How to compile emergency plan and drill for public health emergencies of the infectious diseases. II-2-2 Treatment and prevention to SARS, Ebola hemorrhagic fever and COVID-19 was revised to How to respond to public health of the infectious diseases such as SARS, Ebola hemorrhagic fever and COVID-19. III-2-1 Collection of the infectious disease patients’ blood culture specimen was revised to How to collect different specimen from infectious disease patients and transport blood culture specimen. IV-1-2 Coping with vocational stress in nursing was revised to How to adjust and cope with vocational stress in infectious disease nursing. IV-1-4 Prevention and coping with disputes in nursing was revised to Nursing and corresponding laws. IV-2-2 Promoting psychological healthcare for infectious disease patients from the perspective of positive psychology was revised to psychological nursing of infectious disease patients, and V-1-2 disinfection to infectious diseases was revised to How to disinfect the environment and instrument in infectious disease section.

I-2-4 Management of refined nursing and patients’ safety in infectious disease section was split into How to conduct refined nursing management and How to carry out safety management for infectious disease patients.

How to analyze key nationwide infectious diseases and epidemic trends, How to predict and recognize public health emergencies of the infectious diseases, How to compile emergency plan and drill for public health emergencies of the infectious diseases and Laws and regulations related to major infectious diseases were integrated into The ability of predicting epidemics and conducting emergency drills. How to respond to public health of the infectious diseases such as SARS, Ebola hemorrhagic fever and COVID-19 was integrated into The ability of responding to infectious disease epidemics.

I-1-1 Research progress of infectious disease nursing was deleted on the principle that the item should be deleted if its average was lower than 3.5 and variable coefficient was higher than 0.25. How to apply research project and declare project achievement, How to cultivate scientific research literacy, How to solve clinical issues and transform patent, How to manage nursing personnel in infectious disease section, How to manage materials such as drugs, consumable items and instruments in infectious disease section, How to carry out clinical teaching and practical teaching, How to train nurses in general department under the circumstance of infectious disease emergencies, How to predict and recognize public health emergencies of the infectious disease, Laws and regulations related to major infectious diseases and Nursing care to patients with multiple resistant bacteria were added to the system.

In the second round of questionnaire consultation, 4 experts put forward their suggestions on the revision of the course system: revising 1 class content and 4 class objectives. The specific modifications were as follows. How to analyze key nationwide infectious diseases and epidemic trends was revised to How to analyze key worldwide infectious diseases and epidemic trends while its class objective was revised as “to know the epidemic distribution of the worldwide infectious disease and its trends”. The objective “to master the principles of dosage and route, method of common medication” was revised as “to master the principles of dosage, route and method of common medication, observation of curative effect, and precaution of toxic and side effect”. The objective “to know the nursing care to patients with virus infectious diseases such as mumps, Acquired Immune Deficiency Syndrome, epidemic encephalitis B, hand-foot-and-mouth disease and rabies” was revised as “to know the nursing care to patients with virus infectious diseases such as mumps, Acquired Immune Deficiency Syndrome, epidemic encephalitis B, hand-foot-and-mouth disease, rabies and HFRS”. The objective “to master correct put-on and removal procedures of protective equipment” was revised as “to master the selection of medical protective equipment under different protection levels and correct put-on and removal procedures of protective equipment”.

Finally, core competence training courses for infectious diseases specialist nurses focused on 5 competence modules (professional development abilities, responsiveness to infectious diseases, nursing abilities for infectious diseases, professionalism and humanistic accomplishment, and infection prevention and control abilities). The courses were divided into 12 categories and composed of 66 classes, as shown in Table 8.

**Table 8:** Consultation result of the training course system of infectious disease specialist nurses’ core competence

Competence modules, course categories and class contents (class objectives)	Average	Standard deviation	Variable coefficient	Weighting target
I Professional Development Abilities	4.524	0.512	0.113	0.190
I-1 The ability to do scientific researches on nursing	4.524	0.512	0.113	0.081
I-1-1 How to select and design scientific researches on nursing (to master the principles of research topic selection and the basic principles of topic design)	4.619	0.498	0.108	0.015
I-1-2 How to do literature retrieval and literature quality evaluation (to master Chinese and English language literature retrieval methods, literature management and literature quality evaluation)	4.571	0.507	0.111	0.015
I-1-3 How to prepare questionnaire and select data (to master the principles of questionnaire preparation, methods of electronic questionnaire designing and data collection)	4.714	0.463	0.098	0.015
-1-4 Medical statistics (to master the common medical statistical methods and the methods of filling in and drawing statistical charts so as to perform basic statistical analysis)	4.619	0.498	0.108	0.015
I-1-5 How to write papers and submit papers for publication (to master the requirements and specifications of paper writing and to know the procedures of paper submission)	4.810	0.402	0.084	0.016
I-1-6 How to apply research project and declare project achievement (to know the steps and procedures of research project and achievement declaration)	4.476	0.512	0.114	0.014
I-1-7 How to cultivate scientific research literacy (to master the academic moral norms and medical scientific research ethics, and the methods to improve scientific research)	4.429	0.507	0.115	0.014
I-1-8 How to solve clinical issues and apply patents to clinical practice (to know the existing clinical issues, be able to creatively solve the issues and apply patents to clinical practice in time)	4.381	0.498	0.114	0.014
I-2 The ability of nursing administration	4.571	0.507	0.111	0.082
I-2-1 How to supervise the nursing quality in infectious disease section under the model of responsibility system for nursing (to master the methods of nursing quality management)	4.667	0.483	0.104	0.015
I-2-2 How to conduct refined nursing management (to master the methods of refined nursing and be able to carry out refined nursing according to the patient’s individual difference)	4.714	0.463	0.098	0.015
I-2-3 How to carry out safety management for infectious disease patients (to master the methods and measures of safety management for infectious disease patients)	4.667	0.483	0.104	0.015
I-2-4 How to manage nursing personnel in infectious disease section (to know the management requirements and methods of nursing personnel in infectious disease section)	4.762	0.436	0.092	0.015
I-2-5 How to manage materials such as drugs, consumable items and instruments in infectious disease section (to know the requirements and methods of material management in infectious disease section)	4.714	0.463	0.098	0.015
I-2-6 Procedure and management of clinical reception in fever clinic and enteric diseases clinic (to master the precautions and management of clinical reception for fever clinics and enteric diseases clinics)	4.667	0.483	0.104	0.015
I-2-7 How to manage in operating room in infectious disease hospital or infectious disease department (to know the operating room management procedures and methods in infectious disease hospital or infectious disease department)	4.810	0.402	0.084	0.016
I-3 The ability to conduct nursing education	4.381	0.498	0.114	0.079
I-3-1 How to design classes and conduct teaching methodology (to master the principles and methods of nursing course design and know the different types of teaching methods, such as flipped class model)	4.476	0.512	0.114	0.014
I-3-2 How to make multi-media teaching courseware (to master the techniques and methods of making multi-media courseware)	4.619	0.498	0.108	0.015
I-3-3 How to carry out clinical teaching and practical teaching (to master the principles and requirements for clinical teaching and practical teaching)	4.667	0.483	0.104	0.015
I-3-4 How to train nurses in general department under the circumstance of infectious disease emergencies (to master the content of process-oriented training for nurses in general department under the circumstance of infectious disease emergencies)	4.857	0.359	0.074	0.016
II Responsiveness to Infectious Diseases	4.810	0.402	0.084	0.202
II-1 The ability of predicting epidemic and conducting emergency drills	4.429	0.507	0.115	0.080
II-1-1 How to analyze key worldwide infectious diseases and epidemic trends (to know the distribution of the worldwide infectious diseases and their future trends)	4.571	0.507	0.111	0.015
II-1-2 How to predict and recognize public health emergencies of the infectious diseases (to master how to predict and recognize the public health emergencies of the infectious diseases)	4.619	0.498	0.108	0.015
II-1-3 How to compile emergency plan and drill for public health emergencies of the infectious diseases (to master the response plan for public health emergencies of infectious diseases and carry out emergency drill)	4.714	0.463	0.098	0.015
II-1-4 Laws and regulations related to major infectious diseases (to know the laws and regulations related to major infectious diseases)	4.571	0.507	0.111	0.015
II-2 The ability of responding to infectious disease epidemic	4.714	0.463	0.098	0.085
II-2-1 How to respond to public health of the infectious diseases such as SARS, Ebola hemorrhagic fever and COVID-19 (to accumulate the nursing experiences of responding to major infectious disease events and draw lessons for future improvement)	4.857	0.359	0.074	0.016
III Nursing Abilities for Infectious Diseases	4.857	0.359	0.074	0.204
III-1 The ability of disease nursing	4.810	0.402	0.084	0.086
III-1-1 Introducing to infectious disease nursing (to get an overview on infectious disease nursing)	4.810	0.402	0.084	0.016
III-1-2 Nursing care to infectious disease patients with common symptoms and signs (to master the nursing care to infectious disease patients with common symptoms and signs such as fever, rash, coughing, diarrhea, tic and convulsion)	4.714	0.463	0.098	0.015
III-1-3 Commonly used drugs and nursing care in infectious disease department (to master the principles of dosage, route and method of common medication, observation of curative effect, and precaution of toxic and side effect)	4.667	0.483	0.104	0.015
III-1-4 Nursing care to patients with the flu (to master the nursing care to influenza, Influenza A virus subtype H7N9, and Influenza A virus subtype H1N1)	4.714	0.463	0.098	0.015
III-1-5 Nursing care to patients with rash (to know the nursing care to patients with chicken pox, herpes zoster, measles, rubella, scarlet fever, Epstein-Barr virus, hemorrhagic fever with renal syndrome, scrubtyphus, tsutsugamushi disease, epidemic cerebrospinal meningitis and dengue fever)	4.714	0.463	0.098	0.015
III-1-6 Nursing care to patients with liver diseases (to master the nursing care to patients with acute viral hepatitis, chronic hepatitis, cirrhosis of the liver, hepatic encephalopathy, liver cancer, hepatic failure, upper gastrointestinal hemorrhage and autoimmune liver disease, and patients after transjugular intrahepatic portosystemic shunt and endoscopic esophageal varices ligation)	4.762	0.436	0.092	0.015
III-1-7 Nursing care to patients with virus infectious diseases (to know the nursing care to patients with virus infectious diseases such as mumps, Acquired Immune Deficiency Syndrome, epidemic encephalitis B, hand-foot-and-mouth disease, rabies and Hemorrhagic Fever with Renal Syndrome)	4.619	0.498	0.108	0.015
III-1-8 Nursing care to patients with bacterial infectious diseases (to know the nursing care to patients with bacterial infectious diseases such as bacterial food poisoning, bacillary dysentery, tuberculosis, epidemic cerebrospinal meningitis and brucellosis)	4.619	0.498	0.108	0.015
III-1-9 Nursing care to patients with infectious diseases of protozoa and helminths (to know the nursing care to patients with infectious diseases of protozoa and helminths such as kala-azar, ancylostomiasis, amoebiasis and etc.)	4.667	0.483	0.104	0.015
III-1-10 Nursing care to patients with multiple resistant bacteria (to understand the nursing care to patients with multiple resistant bacteria)	4.667	0.483	0.104	0.015
III-1-11 Treatment to septic shock patients (to master the symptoms and signs of septic shock patients, emergency measures, nursing measures and apply the measures in a flexible manner)	4.667	0.483	0.104	0.015
III-1-12 Nursing care to infectious disease patients during peri-operative period (to master the nursing procedures, complication prevention and precaution for infectious disease patients during peri-operative period)	4.667	0.483	0.104	0.015
III-1-13 Application of clinical pathway in the nursing care for infectious disease patients (to know the common clinical nursing pathway in infectious disease nursing)	4.714	0.463	0.098	0.015
III-2 Common diagnosis and treatment techniques in infectious disease department	4.619	0.498	0.108	0.083
III-2-1 How to collect different specimen from infectious disease patients and transport blood culture specimen (to master methods of specimen collection and precautions of specimen transport, to master the indications, operational approaches and precautions of blood culture from infectious disease patients)	4.762	0.436	0.092	0.015
III-2-2 How to nurse infectious disease patients after paracentesis (to master the nursing techniques for infectious disease patients after liver biopsy, abdominocentesis, lunbar puncture and arterial cannulation)	4.762	0.436	0.092	0.015
III-2-3 How to apply blood purification technology (dialysis or other forms of renal replacement therapy) in infectious disease patients nursing (to master the operational principles and nursing key points of blood purification in infectious disease patients)	4.714	0.463	0.098	0.015
III-2-4 How to deal with endoscopic hemostasis for infectious disease patients (to master the nursing care of endoscopic hemostasis and precautions after operation)	4.667	0.483	0.104	0.015
III-2-5 How to apply respirators among infectious disease patients and nurse artificial airway (to master the usage of respirators, artificial airway nursing and precautions)	4.714	0.463	0.098	0.015
III-2-6 How to nurse infectious diseases patients after compression hemostasis placed with Sengstaken-Blakemore tube (to master the indications of compression hemostasis placed with Sengstaken-Blakemore tube, the nursing interventions, prevention and treatment for complications)	4.714	0.463	0.098	0.015
III-2-7 Standard use of instruments such as infusion pump, injection pump, temperature control blanket and air disinfector in infectious disease section (to master the using steps and precautions of infusion pump, injection pump, temperature control blanket and air disinfector in infectious disease section)	4.810	0.402	0.084	0.016
III-3 Critical thinking ability	4.571	0.507	0.111	0.082
III-3-1 How to apply critical thinking in infectious disease nursing (to master the application of critical thinking in infectious disease nursing)	4.905	0.301	0.061	0.016
III-3-2 How to apply evidence-based nursing in infectious disease nursing (to understand the application of evidence-based nursing in guiding infectious disease practices)	4.857	0.359	0.074	0.016
IV Professionalism and Humanistic Accomplishment	4.667	0.483	0.104	0.196
IV-1 Professionalism	4.429	0.507	0.115	0.080
IV-1-1 How to plan the career (to know nurses’ career planning)	4.667	0.483	0.104	0.015
IV-1-2 How to adjust and cope with vocational stress in infectious disease nursing (to understand the sources of occupational stress in infectious disease nursing work, and master the methods of psychological regulation and stress adjustment)	4.857	0.359	0.074	0.016
IV-1-3 Nurse-patient communication skills (to master the nurse-patient communication skills and principles, and the measures to deal with nurse-patient disputes)	4.571	0.507	0.111	0.015
IV-1-4 Nursing and corresponding laws (to understand the relevant legal knowledge in the infectious disease nursing)	4.429	0.507	0.115	0.014
IV-2 Humanistic care	4.571	0.507	0.111	0.082
IV-2-1 Psychological characteristics of infectious disease patients (to know the infectious disease patients’ negative emotion such as self-abasement and anxiety and their psychological characteristics)	4.381	0.498	0.114	0.014
IV-2-2 Psychological nursing of infectious disease patients (to master the methods of positive psychological nursing and narrative nursing so as to provide psychological nursing to infectious disease patients)	4.619	0.498	0.108	0.015
IV-2-3 Humanistic care in nursing (to master the mode and method of humanistic care in nursing and application of narrative nursing)	4.667	0.483	0.104	0.015
IV-2-4 Health education and health promotion (to master the methods of health education and health promotion so as to carry out health education among infectious disease patients and the public)	4.571	0.507	0.111	0.015
IV-2-5 Application of hospice care among infectious disease patients and their families (to know the application of hospice care among infectious disease patients and their families)	4.381	0.498	0.114	0.014
V Infection Prevention and Control Abilities	5.000	0.000	0.000	0.210
V-1 Disinfection and Quarantine	5.000	0.000	0.000	0.090
V-1-1 How to prevent and control infection in infectious disease section (to master infection prevention and control methods in infectious disease section)	4.905	0.301	0.061	0.016
V-1-2 How to disinfect the environment and instrument in infectious disease section (to master the purpose and types of disinfection, the common disinfection methods and disposal of special infectious disease pollutants)	4.857	0.359	0.074	0.016
V-1-3 Isolation management of infectious diseases (to master the isolation requirements, principles and management system and commonly used isolation technologies)	4.857	0.359	0.074	0.016
V-1-4 How to transport infectious disease patients (to master the methods and protective measures of transport, and the terminal disinfection after transportation)	4.905	0.301	0.061	0.016
V-1-5 How to conduct hand hygiene (to master disinfection approaches of hand hygiene, indications for hand washing, monitoring and requirements of hand hygiene)	4.857	0.359	0.074	0.016
V-1-6 How to deal with medical waste (to master the key points of medical waste classification and collection, transport and temporary storage management in infectious disease section)	4.857	0.359	0.074	0.016
V-1-7 How to monitor and report healthcare-associated infections in hospitals (to master the monitoring scope, reporting, time and process of infectious disease cases in the hospital)	4.905	0.301	0.061	0.016
V-2 Occupational protection	5.000	0.000	0.000	0.090
V-2-1 How to respond to occupational exposure to infectious diseases and corresponding treatment (to know the classification and prevention of vocational exposure to infectious diseases, the evaluation, response and treatment process after vocational exposure to infectious diseases)	4.857	0.359	0.074	0.016
V-2-2 Standard prevention and different types of prevention (to master the basic characteristics, categories and main measures of standard prevention and different types of prevention)	4.810	0.402	0.084	0.016
V-2-3 Management and protection requirements for severe infectious disease section (to know the setting of severe infectious disease section, personnel and material flow, prevention principles and measures)	4.857	0.359	0.074	0.016
V-2-4 Put-on and removal procedures of the medical protective equipment and choose the right personal protective equipment based on a risk analysis on a case-by-case basis (to master the selection of medical protective equipment under different protection levels and correct put-on and removal procedures of protective equipment)	4.810	0.402	0.084	0.016

#### Application of the core competence course training system for infectious disease specialist nurses

The general information of infectious disease specialist nurses in the two groups were shown in Table 9. The general demographic baseline data of the two groups were balancing and comparable.

**Table 9:** General information of infectious disease specialist nurses in two groups (*n* = 105)

Items	The control group(%) (*N* = 50)	The observation group(%)(*N* = 55)	χ^2^	P
Age			0.590	0.745
<30	18	23		
30–40	29	30		
>40	3	2		
Working years			0.771	0.680
<5	10	9		
5–10	23	30		
>10	17	16		
Educational background			0.689	0.708
College certificate	11	16		
Bachelor degree	38	38		
Master degree and above	1	1		
Professional title			0.346	0.841
Nurse or nurse practitioner	30	34		
Nurse in charge	19	19		
Associate chief nurse and above	1	2		
Post			0.122	0.727
Head nurse	2	3		
Nurse	48	52		

Before the training, the core competence of the infectious disease specialist nurses between the two groups had no statistical significance. After the training, the scores in the observation group were significantly higher than those in the control group (*P* < 0.05). Comparing the nurses in the same group before and after the training, the observation group demonstrated significant improvements in professional development abilities, infection prevention and control abilities, nursing abilities for infectious diseases, and responsiveness to emergency infectious diseases (*P* < 0.05). These results indicate that the training courses are both scientifically designed and effective, as depicted in Table 10.

**Table 10:** Comparison of core competence differences between the observation group and the control group

	Before the training		t	P	After the training		t	P
	The control group	The observation group			The control group	The observation group		
Infectious disease specialist nurse’s core competence	136.42 ± 26.32	138.18 ± 19.19	-0.394	0.694	139.82 ± 10.76	148.16 ± 9.88*	-2.719	0.008
Professional Development Abilities	39.98 ± 10.38	40.47 ± 7.57	-0.280	0.780	41.84 ± 6.22	45.02 ± 5.76*	-2.447	0.016
Infection Prevention and Control Abilities	38.90 ± 6.71	39.05 ± 5.67	-0.128	0.899	39.58 ± 4.08	41.38 ± 3.46*	-3.512	0.001
Nursing Abilities for Infectious Diseases	24.12 ± 4.98	24.85 ± 3.88	-0.847	0.399	24.74 ± 2.78	26.40 ± 2.03*	-2.068	0.041
Professionalism and Humanistic Accomplishment	21.32 ± 3.81	21.75 ± 3.04	-0.635	0.527	21.38 ± 1.88	22.25 ± 2.39	-2.881	0.005
Responsiveness to Emergency Infectious Diseases	12.10 ± 2.72	12.05 ± 2.34	0.092	0.927	12.28 ± 1.67	13.11 ± 1.27*	-4.142	< 0.01

*Notes*: * indicated the differences of the same group before and after the training (*P*<0.05)

## Discussion

### Reliability and scientific validity of Delphi expert consultation results

Under the guidance of the previous index system of core competence assessment for infectious disease specialist nurses [19] and on the basis of fully reviewing the core competence training course of specialist nurses [24–26] and core competence theory [27–29], after rigorous Delphi expert consultation, this study constructed the training course system for infectious disease specialist nurses. The system contained 4 aspects, such as the training competence modules, course categories, class contents and class objectives.

Before class contents were formulated, the research group held group meeting, consulted and drew feedback from the experts, and invited them to assess the feasibility of the first draft of the course system so as to ensure the practicability of the class contents. The experts involved in the research belonged to level-A tertiary hospitals, infectious disease specialist hospitals and School of Nursing in high educational institutions in 5 provinces and cities around China. They all had rich clinical and nursing education experience of infectious diseases. The Kendall’s coefficient of concordance of the two rounds of Delphi expert consultation after significance testing (*P* < 0.05) indicated that the experts’ opinion coordination degree was preferable and the results of the study were reliable. As there was no standardized training course system of core competence of infectious disease specialist nurses, our study scientifically constructed a comprehensive training course system.

### Analysis of course system contents

The training course system of in this study focused on 5 competence modules and was divided into 12 course categories which involved 66 class contents and corresponding objectives. The competence module of professional development was the key point of this training course system. Scientific research on nursing was the biggest problem that had always plagued the clinical nurses [30, 31]. Compared with ordinary nurses, the specialist nurses should have a more systematic and comprehensive scientific research knowledge system and capability [32]. Besides, a high level of scientific research could improve efficiency and quality of nursing [33]. Our study targeted on the weaknesses of clinical nurses and problems that urgently needed to be solved. Scientific research on nursing was regarded as the priority of the training course system. The system not only covered such contents as scientific research selection and design, literature review and statistical analysis and paper writing, but also extended to the related contents such as research project application and achievement declaration, clinical issues and patent transformation so that the system could effectively guarantee the improvement of the specialist nurses’ core competence. Although scientific research is important to nursing practices, it should not be the sole focus or priority of training and education programs which should virtually focus on ability development and practical approaches. In the competence modules of professional development, the weighting target of classes of nursing management accounted for the largest proportion, around 0.082. It covered the contents of management of nursing quality, management of refined nursing and so on, aiming to improve the nursing management capabilities of the infectious disease specialist nurses through comprehensive management training classes so that the specialist nurses could play the role of nursing managers in clinical work and improve the quality of infectious disease nursing. Specialist nurses not only needed to play such important roles as nursing staff and nursing managers, but also needed to act as nursing educators who could deliver lectures on infectious diseases to ordinary nurses, nursing students and nursing staff in other departments [34, 35]. In response to the COVID-19 pandemic, nurses in infectious disease department proffered great help to nurses in other departments in quickly responding to the pandemic [36].

The infectious disease specialist nurses should not only be proficient in infectious disease nursing, but also have the ability to deal with infectious diseases. Considering the infectious disease specialist nurses lacked the ability of response to infectious diseases, the competence module of response to infectious disease was regarded as a relatively important part of this training course system. Starting with the two course categories, including prediction and emergency drills and response to infectious disease epidemics, this module elaborated on the distribution of the current key global infectious diseases and analysis of their development trend, prediction and recognition of the public health emergencies, interpretation of laws and regulations related to major infectious diseases, and China’s experience in responding to major outbreaks of infectious diseases in the past ten years. The contents of this module ranged from theory to practice, and from macro analysis to specific operations. This kind of setting aimed to yield effective measures and meet the needs of responding to future infectious disease emergencies.

Nursing abilities for infectious disease were the basic abilities that infectious disease specialist nurses should acquire. The weighting target of this competence module was 0.204, ranking the second. This competence module of nursing for infectious disease included three categories, namely nursing capabilities, commonly used diagnosis and treatment technology in infectious disease department and critical thinking capabilities, with nursing capabilities accounting for the largest proportion of 0.086. Infectious disease specialist nurses should be proficient in the symptoms and signs of different types of infectious disease patients and corresponding nursing skills, and should take targeted nursing measures so as to provide patients with high-quality nursing service [20]. They should also be proficient in commonly used diagnosis and treatment techniques in infectious disease nursing process. At the same time, they should foster their critical thinking capability in the process of training [37], which was important in nursing work because it could help avoid nursing errors and accidents and improve work efficiency. The classes of critical thinking aimed to strengthen the specialist nurses’ critical thinking abilities in the nursing of clinical infectious disease.

The competence module of professionalism and humanistic accomplishment consisted of two categories, professionalism and humanistic care, with respective weightings of 0.080 and 0.082. The classes of professionalism aimed at improving the infectious disease specialist nurses’ professionalism through the study of career planning and vocational stress coping in infectious disease nursing. The classes of humanistic accomplishment encompassed humanistic care in nursing, health education and health promotion, and application of hospice among infectious disease patients. The classes reflected the humanistic feelings in infectious disease nursing, including the care to infectious disease patients and the health education to the public, as well as the hospice care to infectious disease patients at the terminal stage.

The competence module of infection prevention and control made up the largest proportion of the expert questionnaire consultation in this training course system. Infection prevention and control ability was the most important ability which affected the continuation of the combat effectiveness of nursing staff. The competence module of infection prevention and control included two categories, disinfection and quarantine, and occupational protection. These categories had equal weighting targets, accounting for approximately 0.090 each. The disinfection and quarantine classes, as well as the occupational protection classes, were structured to cover both theoretical and practical aspects so as to provide comprehensive lectures on infectious disease disinfection and quarantine, improvement and standardization of infectious disease specialist nurses’ occupational protection ability.

### Significance of the training course system

At present, great differences lie in training contents, objectives, resources and management among infectious disease specialist nurses. Our study established a comprehensive, scientific and standardized training course system which covered both the competence requirements that general nurses need to meet and the contents that infectious disease specialist nurses should learn. The study has practical significance in training specialist nurses and especially in improving both the overall training quality and core competence of infectious disease specialist nurses. Homogeneous training of infectious disease specialist nurses plays an important role in responses to public health emergencies of major infectious diseases. If nurses get homogeneous training with the uniform standards and nursing procedures, they can definitely cooperate better and fulfil their work faster when they gather together from different provinces and cities to fight against the epidemics. In this study, the training course system has been developed through group discussions and expert consultations to better meet the expectations of infectious disease specialist nurses. Through training course system, infectious disease specialist nurses can systematically learn the professional knowledge, master new nursing skills and techniques, and improve the accuracy and proficiency of actual practice. And it may serve as a reference for the homogeneous training of infectious disease specialist nurses in the future.

## Limitations

There are some limitations in the construction of the training course system of core competence of infectious disease specialist nurses by way of Delphi expert consultation. The Delphi study is prone to subjective factors and may lack profound theoretical and logical arguments. Besides, it lasts for a long period of time. Although the expert participants were selected from three different fields such as infectious disease health care, infectious disease nursing and infectious disease nursing education, and from five different provinces and cities, it would be more representative if the experts belonged to more fields, and more provinces and cities. In the subsequent study, we will explore the duration of the acquisitions and implement the training course in diverse geographical contexts.

## Conclusion

In this study, through two rounds of Delphi expert questionnaire consultation, we establish the training course system of core competence for infectious disease specialist nurses which focuses on 5 competence modules and consists of 12 course categories with 66 class contents and corresponding objectives. On the basis of the scientific and rigorous research methods, as well as the comprehensive content of the training course system, we have obtained the reliable research results. In conclusion, the training course system can serve as a valuable reference for training infectious disease specialist nurses.

## Data Availability

The datasets generated and analyzed during the current study are not publicly available due to the protection of the privacy of consulting experts but are available from the corresponding author (906963251@qq.com) on reasonable request.
